# Sphingomyelin Synthase 1 (SMS1) Downregulation Is Associated With Sphingolipid Reprogramming and a Worse Prognosis in Melanoma

**DOI:** 10.3389/fphar.2019.00443

**Published:** 2019-04-30

**Authors:** Fatima Bilal, Anne Montfort, Julia Gilhodes, Virginie Garcia, Joëlle Riond, Stéphane Carpentier, Thomas Filleron, Céline Colacios, Thierry Levade, Ahmad Daher, Nicolas Meyer, Nathalie Andrieu-Abadie, Bruno Ségui

**Affiliations:** ^1^INSERM UMR 1037, CRCT, Toulouse, France; ^2^Equipe Labellisée Ligue Contre Le Cancer, Toulouse, France; ^3^Ecole Doctorale de Sciences et Technologies, Université Libanaise, Beirut, Lebanon; ^4^Université Toulouse III – Paul Sabatier, Toulouse, France; ^5^Institut Universitaire du Cancer, Toulouse, France; ^6^Laboratoire de Biochimie, CHU Purpan, Institut Fédératif de Biologie, Toulouse, France

**Keywords:** sphingolipids, ceramide, glucosylceramide, prognosis biomarker, cancer

## Abstract

Sphingolipid (SL) metabolism alterations have been frequently reported in cancer including in melanoma, a bad-prognosis skin cancer. In normal cells, *de novo* synthesized ceramide is mainly converted to sphingomyelin (SM), the most abundant SL, by sphingomyelin synthase 1 (SMS1) and, albeit to a lesser extent, SMS2, encoded by the *SGMS1* and *SGMS2* genes, respectively. Alternatively, ceramide can be converted to glucosylceramide (GlcCer) by the GlcCer synthase (GCS), encoded by the *UGCG* gene. Herein, we provide evidence for the first time that SMS1 is frequently downregulated in various solid cancers, more particularly in melanoma. Accordingly, various human melanoma cells displayed a SL metabolism signature associated with (i) a robust and a low expression of *UGCG* and *SGMS1/2*, respectively, (ii) higher *in situ* enzyme activity of GCS than SMS, and (iii) higher intracellular levels of GlcCer than SM. SMS1 was expressed at low levels in most of the human melanoma biopsies. In addition, several mutations and increased CpG island methylation in the *SGMS1* gene were identified that likely affect SMS1 expression. Finally, low SMS1 expression was associated with a worse prognosis in metastatic melanoma patients. Collectively, our study indicates that SMS1 downregulation in melanoma enhances GlcCer synthesis, triggering an imbalance in the SM/GlcCer homeostasis, which likely contributes to melanoma progression. Evaluating SMS1 expression level in tumor samples might serve as a biomarker to predict clinical outcome in advanced melanoma patients.

## Introduction

Melanoma is the most dangerous and deadliest form of skin cancers. Despite emerging targeted therapies and immunotherapies, most of the patients do not respond optimally, and/or develop acquired resistance (Eroglu and Ribas, 2016; Sharma et al., 2017).

Sphingolipids (SL) are bioactive molecules that play key roles in plasma membrane homeostasis and dynamics as well as in many cellular processes including cell death and proliferation as well as cancer progression (Hannun, 1996; Hannun and Obeid, 2002; Ogretmen and Hannun, 2004; Segui et al., 2006). In melanoma, numerous studies have documented alterations in SL metabolism (Colie et al., 2009; Sorli et al., 2013; Albinet et al., 2014; Mrad et al., 2016; Leclerc et al., 2018). Glucosylceramide synthase (GCS), which converts ceramide to glucosylceramide (GlcCer), is involved in melanoma progression in mice (Deng et al., 2002; Weiss et al., 2003). To the best of our knowledge, sphingomyelin synthases SMS1 and SMS2 (encoded by the *SGMS1* and *SGMS2* genes), which metabolize ceramide into sphingomyelin (SM) (Huitema et al., 2004; Yamaoka et al., 2004), the most abundant SL in mammalian cells (Lafont et al., 2010), have not been analyzed in melanoma.

Herein, we show that SMS1 downregulation (i) occurs frequently in melanoma, (ii) is associated with SL reprogramming, and (iii) constitutes a worse prognosis biomarker in metastatic melanoma.

## Materials and Methods

### Macroarray Experiment

Cancer Profiling array II (#631777) including patient-derived cDNA tumor and non-tumor samples was purchased from BD Biosciences Clontech. Human samples were collected in accordance with all applicable laws and regulations in an ethical manner. Membrane was successively hybridized according to the manufacturer’s instructions with SMS1 and ubiquitin ^32^P-labeled probes generated using a random nonamer primer labeling procedure (# RPN1604, Amersham Biosciences). The membrane was exposed to an intensifying screen that was developed using PhosphorImager and Image Quant software.

### *SGMS1*, *SGMS2*, and *UGCG* Expression and Mutations in Human Melanoma

*SGMS1*, *SGMS2*, and *UGCG* expression was evaluated from Oncomine database (Haqq et al., 2005; Talantov et al., 2005; Riker et al., 2008) and the cancer genome atlas (TCGA) melanoma (Cancer Genome Atlas Network, 2015). TCGA genomic and clinical data were downloaded from the UCSC cancer genome browser project^1^. The analysis population consisted of 342 patients with distant metastasis for whom RNAseq and clinical data overlap. All survival times were calculated from the date of specimen procurement and were estimated by the Kaplan Meier method with 95% confidence intervals (CI). Univariates analyses were performed using Cox proportional hazards model. Alternatively, *SGMS1, SGMS2, and UGCG* mutation analyses in human melanoma were assessed on cBioportal^2^ (Cerami et al., 2012; Gao et al., 2013).

### *SGMS1* Methylation Analysis

The correlation between *SGMS1* expression and methylation status of *SGMS1* CpGs in metastatic patient samples was analyzed using the TCGA melanoma RNA-seq and DNA methylation Illumina datasets. For each analyzed CpG, the rho values, indicating the Spearman’s rank correlation coefficients between the CpG methylation and the *SGMS1* expression, are reported. The organization of the *SGMS1* locus is depicted in Figure 2A as previously described (Vladychenskaya et al., 2004).

### Melanoma Cell Lines

Human melanoma cell lines (M249, SKMEL28, A375, WM9, WM35, WM115, WM266, WM793, WM1346, COLO829, and G361) were from ATCC or Wistar institute.

### Determination of *in situ* SMS and GCS Activities

1 × 10^6^ melanoma cells were incubated with 2.5 μM C6-NBD-ceramide (Sigma) solubilized in ethanol and SMS and GCS activities were measured as previously described (Lafont et al., 2010; Bilal et al., 2017a).

### Analysis of Sphingolipids

Sphingolipids were analyzed from 1.10^6^ melanoma cells by liquid chromatography/mass spectrometry (LC/MS) as previously described (Bilal et al., 2017b).

### qRT-PCR Analysis

Total RNA was reverse-transcribed using 1 μg of input RNA and random primers (SuperScript II, Invitrogen). qRT-PCR reactions were performed in duplicate on StepOne apparatus (Applied Biosystems) using SYBR Green (QuantiTect, Qiagen) as fluorescent detection dye. Results were quantified and mRNA expression for each target gene (*UGCG*, *SGMS1*, or *SGMS2*) was determined by normalization to reference genes (β-actin and GAPDH) using the ΔCt method. Primers for *UGCG* and reference genes were from Qiagen. Primers for *SGMS1* and *SGMS2* were from Sigma (Lafont et al., 2010).

### Statistics and Reproducibility

**Figure 1:**
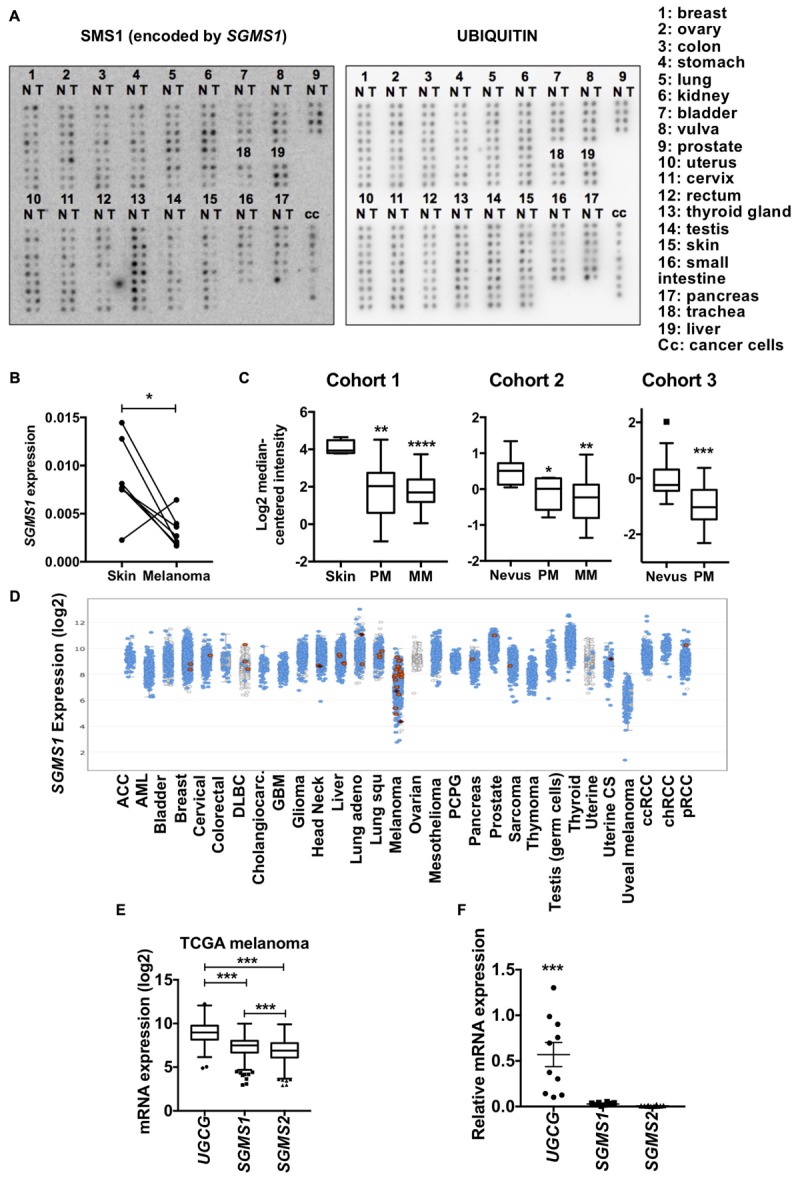
Sphingomyelin synthase 1 (SMS1) is frequently downregulated in melanoma. **(A)** cDNA samples isolated from normal (N) and tumor (T) tissues from the same patient were compared. Expression of *SGMS1* (left panel) and ubiquitin (right panel). **(B)** The *SGMS1* expression was normalized to ubiquitin and expressed for each pair in normal skin and melanoma samples. **(C)** *SGMS1* expression was analyzed in 3 different cohorts from Oncomine in normal Skin (*n* = 4), primary (PM; *n* = 14), and metastatic (MM; *n* = 39) melanoma (Ricker’s cohort) (left panel); in nevus (*n* = 9), primary (PM; *n* = 6), and metastatic (MM; *n* = 19) melanoma (Haqq’s cohort) (middle panel); in nevus (*n* = 18) and primary melanoma (PM; *n* = 45) (Talantov’s cohort) (right panel). **(D)** The expression of *SGMS1* was analyzed in various cancer type cohorts from cbioportal. **(E)** The expression of *UGCG*, *SGMS1*, and *SGMS2* was analyzed in melanoma samples from the TCGA metastatic melanoma patients (*n* = 342). **(F)** A set of melanoma cell lines (*n* = 10) was analyzed for the expression of *UGCG*, *SGMS1*, and *SGMS2* by RT-qPCR (*n* = 10). Data from at least two independent experiments are means ± SEM. ^∗^*p* < 0.05, ^∗∗^*p* < 0.01, ^∗∗∗^*p* < 0.001, and ^∗∗∗^*p* < 0.0001.

Statistical significance of differences between groups was evaluated using the Graph-Pad Prism 7 software. For multiple comparisons, an Anova test was used. Wilcoxon test was used in Figure 1B. Differences were considered to be statistically significant when *p* < 0.05 (^∗^*p* < 0.05; ^∗∗^*p* < 0.01; ^∗∗∗^*p* < 0.001).

## Results

### SMS1 Downregulation in Melanoma Is Associated With SL Metabolism Reprogramming

We initially performed a macroarray to evaluate the expression of SMS1 in matched tumor and non-tumor samples from the same patients (Figure 1A). The data analysis with a threshold of 1.5 showed that, whereas SMS1 was up-regulated in 11% of tumor samples, it was down-regulated in 46% of tumor samples (Supplementary Table 1). As a matter of fact, SMS1 was most frequently down-regulated in vulva (5 out of 5), testis (9 out of 10), and skin (9 out of 10) cancers, including melanoma (6 out of 7) (Figure 1B and Supplementary Table 1). Accordingly, our transcriptomic analysis in 3 different cohorts from published database indicates that *SGMS1* was downregulated in primary and metastatic human melanoma as compared to normal skin and nevus (Figure 1C; Haqq et al., 2005; Talantov et al., 2005; Riker et al., 2008). In contrast, the expression of *SGMS2* and *UGCG*, encoding SMS2 and GCS, respectively, remained unchanged (Supplementary Figure 1A). We next evaluated the expression of *SGMS1* in various cancer types from the TCGA database. Strikingly, melanoma exhibited the lowest expression of *SGMS1* (Figure 1D). Moreover, melanoma expressed *SGMS2* at rather low levels, while expressing *UGCG* at high levels (Supplementary Figure 1B). In metastatic melanoma from the TCGA, the expression of *UGCG* was significantly higher than that of *SGMS1* and *SGMS2* (Figure 1E). Accordingly, melanoma cells exhibited low *SGMS1* and *SGMS2* expression, while they expressed *UGCG* at higher levels (Figure 1F).

**Figure 2:**
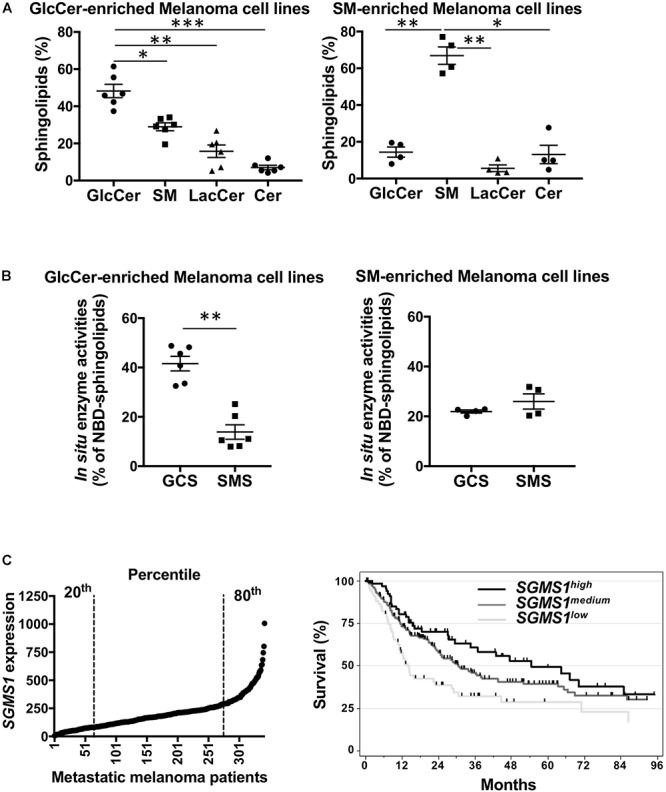
Sphingomyelin synthase 1 downregulation is associated with a worse prognosis in advanced melanoma patients. **(A**,**B)** A set of melanoma cell lines (*n* = 10) was analyzed for SLs by mass spectrometry **(A)** and GCS and SMS enzyme activities **(B)**. Data from one experiment representative of three independent experiments are means ± SEM. **(C)** *SGMS1* expression in melanoma samples from the TCGA melanoma cohort (*n* = 342) (left panel) and overall survival of patients exhibiting low (*n* = 68), medium (*n* = 206), and high (*n* = 68) *SGMS1* expression (right panel). Cox model: *SGMS1^low^* (Reference), *SGMS1^medium^*: HR = 0.62 [95% CI = 0.44; 0.88] *p* = 0.007; *SGMS1^high^*: HR = 0.48 [95% CI 0.31; 0.76] *p* = 0.002. ^∗^*p* < 0.05, ^∗∗^*p* < 0.01, and ^∗∗∗^*p* < 0.001.

We next evaluated the SL metabolism signature in human melanoma cell lines. Whereas four melanoma cell lines exhibited a higher proportion of SM, six were enriched in GlcCer (Figure 2A). Accordingly, *in situ* enzyme activity was significantly higher for GCS than for SMS in the cell lines with high GlcCer proportion only (Figure 2B). Consequently, endogenous intracellular levels of GlcCer were greater than SM and other SL species as evaluated by mass spectrometry for those six melanoma cell lines (Figure 2A and Supplementary Table 2). Of note, neither the mutation status (Bairoch, 2018) nor the origin of the melanoma cell lines (i.e., from radial or vertical growth phase or metastasis) were associated with a specific SL signature (Supplementary Table 3).

### SMS1 Downregulation in Human Melanoma Is Associated With a Worse Prognosis

To get insight into the molecular mechanisms that may account for SMS1 downregulation and/or inhibition of enzyme activity, we evaluated *SGMS1* mutation and methylation status in the public databases of melanoma. Whereas *SGMS2* and *UGCG* were mutated with low frequency, *SGMS1* exhibited a higher mutation rate in the coding sequence (Supplementary Figure 2). Most of the mutations were missense mutations and some of them affected residues in the catalytic domain (Supplementary Figure 2 and Supplementary Table 4). In the TCGA melanoma cohort, 7.7% of the 287 sequenced samples were mutated (16 missense mutations, 2 non-sense mutations and 4 deep deletions). One of the non-sense mutations (W309^∗^) was also found in one specimen from another melanoma cohort (Supplementary Table 4). The other non-sense mutation (R387^∗^) was also found in colorectal carcinoma, sarcoma and uterus carcinoma (data not shown).

To delineate the effect of DNA methylation on the regulation of *SGMS1* expression, we analyzed the TCGA metastatic melanomas. Among the 50 *SGMS1* Illumina 450K probes with workable data, the DNA methylation level of 33 probes displayed a significant correlation with expression. Ten probes out of the 14 located in the CpG island 1 and its shores, as well as 3 out of the 3 in the CpG island 2 and its shores, both containing putative promoter sequences, were inversely correlated with the expression level. In contrast, 13 CpG out of the 16 located outside CpG islands and shores were positively correlated with the expression (Supplementary Figures 3A,B). Thus, hypermethylation of CpG islands and hypomethylation events in open sea were significantly associated with the decrease in *SGMS1* expression, indicating the regulation of SMS1 expression in metastatic melanoma might rely, at least partly, on DNA methylation of the *SGMS1* locus.

Finally, the clinical outcome in metastatic melanoma patients exhibiting low (20th percentile, medium (between the 20th and 80th percentile), high (80th percentile) *SGMS1, SGMS2, and UGCG* expression was analyzed in the TCGA cohort. Whereas *UGCG* and *SGMS2* expression did not impact on overall survival (Supplementary Figure 4), low *SGMS1* expression was statistically associated with shortened overall survival (Figure 2C).

Collectively, our data indicate that melanoma exhibit a SL metabolism reprogramming associated with SMS1 downregulation, which constitutes a worse-prognosis biomarker.

## Discussion

Herein, we provide evidence for the first time that melanoma exhibit SL metabolism changes associated with SMS1 down-regulation, not only decreasing SM synthesis but also promoting the synthesis of GlcCer, which facilitates tumor progression in mouse melanoma models (Deng et al., 2002; Weiss et al., 2003). Interestingly, a recent study indicates the formation of an heterocomplex between SMS1 and GCS in mammalian cells, which enhances and reduces SM, and GlcCer synthesis, respectively (Hayashi et al., 2018). SMS1 downregulation may limit the formation of such a complex, promoting GlcCer synthesis in melanoma. Since *de novo* synthesized ceramide is the substrate of both SMS1 and GCS in the Golgi, downregulation of SMS1 likely increases the ceramide pool available for GCS to produce GlcCer.

Strikingly, low SMS1 expression is associated with a worse prognosis in metastatic melanoma, suggesting that reduced SM synthesis likely contributes to melanoma progression. SMS1 down-regulation, which occurs in primary melanoma, is likely an early event in melanomagenesis. Several mutations affecting the coding sequence of *SGMS1* probably contribute to the decreased SMS1 expression. In the TCGA melanoma cohort, 5 out of 16 missense mutations and 1 non-sense mutation were associated with shallow deletions. Moreover, 4 deep deletions were identified as well as 17 CpG located on the two CpG islands and their shores, the methylation of which was correlated with *SGMS1* downregulation in metastatic melanoma. SMS1 expression and activity are likely regulated by translational and post-translational mechanisms such as recently described in Bcr-Abl-expressing leukemia cells (Moorthi et al., 2018). Whereas key driver mutations have been identified in melanoma (Hodis et al., 2012), we found no correlation between mutation status and SL signature.

Ceramide clearance catalysed by GCS plays a role in multidrug resistance of cancer cell lines (Lavie et al., 1997; Liu et al., 2004; Sun et al., 2006; Liu et al., 2010). However, our team provided genetic evidence that GCS is unlikely a critical enzyme to confer melanoma resistance to chemotherapy in a mouse melanoma model (Veldman et al., 2003). Because SLs are key components of the plasma membrane, modulating various signaling pathways (Hannun, 1996; Hannun and Obeid, 2002), future experiments will address whether or not SM/GlcCer homeostasis alterations in melanoma impair the efficacy of emerging therapies such as targeted therapies and immunotherapies. Finally, it remains to evaluate whether low SMS1 expression in melanoma samples is a valuable biomarker to predict the resistance of patients to emerging therapies.

## Ethics Statement

As clearly stated in the manuscript, the results shown here are part based upon data generated by the TCGA Research Network. In addition, we have analyzed data from the oncomine database. We have performed a macroarray to evaluate the expression of SMS1 in matched tumor and non-tumor samples from the same patients by using the Cancer Profiling array II (Clontech #631777) membrane.

## Author Contributions

FB, VG, and SC performed the experiments. JG and JR performed the methylation analyses. JG and TF performed the bio-statistical analyses of the TCGA melanoma. AM, CC, TL, AD, NM, and NA-A edited the manuscript. SB designed and supervised the study and wrote the manuscript.

## Conflict of Interest Statement

NM has worked as an investigator and/or consultant and/or speaker for: BMS, MSD, Amgen, Roche, GSK, Novartis, and Pierre Fabre. BS has worked as an investigator for BMS. The authors disclose they are in the process applying for a patent based upon these findings. The remaining authors declare that the research was conducted in the absence of any commercial or financial relationships that could be construed as a potential conflict of interest.

## Footnotes

1 https://genome-cancer.ucsc.edu

2 http://www.cbioportal.org/
