# Supplementary material for: Sphingomyelin Synthase 1 (SMS1) Downregulation Is Associated With Sphingolipid Reprogramming and a Worse Prognosis in Melanoma
Source: Front Pharmacol. 2019 Apr 30;10:443. doi: 10.3389/fphar.2019.00443 (PMC6503817; doi:10.3389/fphar.2019.00443)
Supplement: Supplementary file 1 [file Data_Sheet_1.PDF]

Supplementary information

Supplementary Table 1: *SGMS1* gene upregulation and downregulation in cancer tissues of the cancer-profiling array. The extents of *SGMS1* gene upregulation (T/N>1.5) and downregulation (N/T>1.5) observed in each cancer tissue have been averaged (mean fold±s.e.m.).

Tissue	*Breast*	*Ovary*	*Colon*	*Stomach*	*Lung*	*Kidney*	*Bladder*	*Trachea*	*Vulva*	*Liver*
Increase frequency	3/10	4/10	0/10	2/10	0/10	0/10	0/5	1/3	0/5	0/3
Mean fold ±s.e.m.	2.1±0.16	1.68±0.12	-	1.65±0.03	-	-	-	2.23	-	-
Decrease frequency	3/10	2/10	3/10	2/10	6/10	6/10	1/5	1/3	5/5	2/3
Mean fold ±s.e.m.	1.85±0.34	1.66±0.04	1.97±0.12	2.88±0.68	3.07±0.53	2.72±0.25	2.01	2.17	2.55±0.25	2.59±0.82
Tissue	*Prostate*	*Uterus*	*Cervix*	*Rectum*	*Thyroid gland*	*Testis*	*Skin*	*Melanoma*	*Small intestine*	*Pancreas*
Increase frequency	2/4	1/10	1/10	0/10	1/10	0/10	1/10	1/7	1/7	0/7
Mean fold ±s.e.m.	2.9±0.31	2.49	2.07	-	1.53	-	2.84	2.84	1.6	-
Decrease frequency	0/4	4/10	3/10	4/10	5/10	9/10	9/10	6/7	3/7	4/7
Mean fold ±s.e.m.	-	1.94±0.23	1.87±0.16	1.95±0.16	2.31±0.37	3.3±0.51	3.64±0.46	3.94±0.59	2.23±0.35	2.91±0.58

Supplementary Table 2: Sphingolipid content in various melanoma cell lines. Values are pmol of sphingolipids per mg of proteins. Data are from 1 experiment representative of three independent experiments.

**Sphingolipid (pmol/mg)**	**Melanoma cell lines**									
	**Glucosylceramide-enriched cell lines**						**Sphingomyelin-enriched cell lines**			
	**Colo829**	**WM9**	**WM35**	**M249**	**SKMEL28**	**G361**	**WM793**	**WM266**	**A375**	**WM115**
**Ceramide**	1028	1395	1218	549	499	1045	2959	767	745	3079
**Glucosylceramide**	15149	5425	5570	3249	4206	10796	2347	3101	1386	1295
**GM3 ganglioside**	2283	282	937	1323	1427	1688	2823	1203	733	1460
**Lactosylceramide**	1297	829	4001	1572	1426	3795	1418	506	822	380
**Sphingomyelin**	7192	3947	4108	2314	3053	3793	22672	11510	4573	6369

Supplementary Table 3: Mutation status of the melanoma cell lines

Glucosylceramide-enriched melanoma cell lines		
**Cell lines**	**Origin**	**Mutations**
**M249**	Metastasis	Heterozygous for $BRAF^{V600E}$<br>Homozygous for *PTEN* deletion
**SKMel28**	Metastasis	Homozygous for $BRAF^{V600E}$<br>Heterozygous for $CDK4^{R24C}$<br>Homozygous for $EGFR^{P753S}$<br>Homozygous for $PTEN^{T167A}$<br>Homozygous for $TP53^{L145R}$
**WM35**	RGP	Heterozygous for $BRAF^{V600E}$
**WM9**	Metastasis	Heterozygous for $BRAF^{V600E}$<br>Hemizygous for *PTEN* deletion
**COLO829**	Metastasis	Heterozygous for $BRAF^{V600E}$<br>Homozygous for *CDKN2A* deletion<br>Homozygous for *PTEN* deletion
**G361**	VGP	Heterozygous for $BRAF^{V600E}$

Sphingomyelin-enriched melanoma cell lines		
Cell lines	**Origin**	**Mutations**
**WM115**	VGP	Heterozygous for $BRAF^{V600D}$<br>Hemizygous for PTEN deletion
**A375**	Metastasis	Homozygous for $BRAF^{V600E}$<br>Homozygous for $CDKN2A^{E61Ter,\ G75V,\ E69Ter,\ G83V}$
**WM266**	Metastasis	Heterozygous for $BRAF^{V600D}$<br>Hemizygous for PTEN deletion
**WM793**	VGP	Heterozygous for $BRAF^{V600E}$<br>$CDK4^{K22Q}$

Supplementary Table 4: Mutations on *SGMS1* sequence in melanoma. Nonsense mutations are in bold.

Sample ID	Cancer Study	AA change	Type	Copy #
**49M**	DESM (Broad 2015)	F216I	Missense	NA
**MEL-Ma-Mel-65**	Melanoma (Broad)	P162S	Missense	NA
**MEL-Ma-Mel-62**	Melanoma (Broad)	E131K	Missense	NA
MEL-13575	**Melanoma (Broad)**	**W309***	**Nonsense**	**NA**
**MEL-JWCI-WGS-6**	Melanoma (Broad)	G250R	Missense	NA
**MEL-Ma-Mel-102**	Melanoma (Broad)	D327N	Missense	NA
**MEL-JWCI-WGS-1**	Melanoma (Broad)	F393I	Missense	NA
**MEL-JWCI-14**	Melanoma (Broad)	L323F	Missense	NA
TCGA-EE-A3J7-06	**Melanoma (TCGA)**	**W309***	**Nonsense**	**ShallowDel**
**TCGA-D3-A2J8-06**	Melanoma (TCGA)	D43N	Missense	Diploid
**TCGA-EE-A2MR-06**	Melanoma (TCGA)	P168L	Missense	Diploid
**TCGA-EE-A29G-06**	Melanoma (TCGA)	M130I	Missense	ShallowDel
**TCGA-FW-A3R5-06**	Melanoma (TCGA)	D35N	Missense	Diploid
**TCGA-EE-A2MS-06**	Melanoma (TCGA)	R257Q	Missense	Diploid
**TCGA-EE-A20C-06**	Melanoma (TCGA)	P301S	Missense	Diploid
**TCGA-FW-A3R5-06**	Melanoma (TCGA)	S53F	Missense	Diploid
**TCGA-EE-A3J5-06**	Melanoma (TCGA)	E158K	Missense	ShallowDel
**TCGA-D3-A51G-06**	Melanoma (TCGA)	E188K	Missense	ShallowDel
**TCGA-FW-A3R5-06**	Melanoma (TCGA)	E131K	Missense	Diploid
**TCGA-EE-A183-06**	Melanoma (TCGA)	M152I	Missense	Diploid
TCGA-EE-A29B-06	**Melanoma (TCGA)**	**R387***	**Nonsense**	**Diploid**
**TCGA-EE-A183-06**	Melanoma (TCGA)	H308Y	Missense	Diploid
**TCGA-FS-A1ZW-06**	Melanoma (TCGA)	P27L	Missense	ShallowDel
**TCGA-EE-A2MP-06**	Melanoma (TCGA)	I87N	Missense	Diploid
**TCGA-EE-A2MC-06**	Melanoma (TCGA)	M63I	Missense	Diploid
**TCGA-EE-A181-06**	Melanoma (TCGA)	M63I	Missense	ShallowDel
**YUKLAB**	Melanoma (Yale)	P162S	Missense	NA
**YUAKER**	Melanoma (Yale)	L140F	Missense	NA
**YULAPE**	Melanoma (Yale)	S144F	Missense	NA

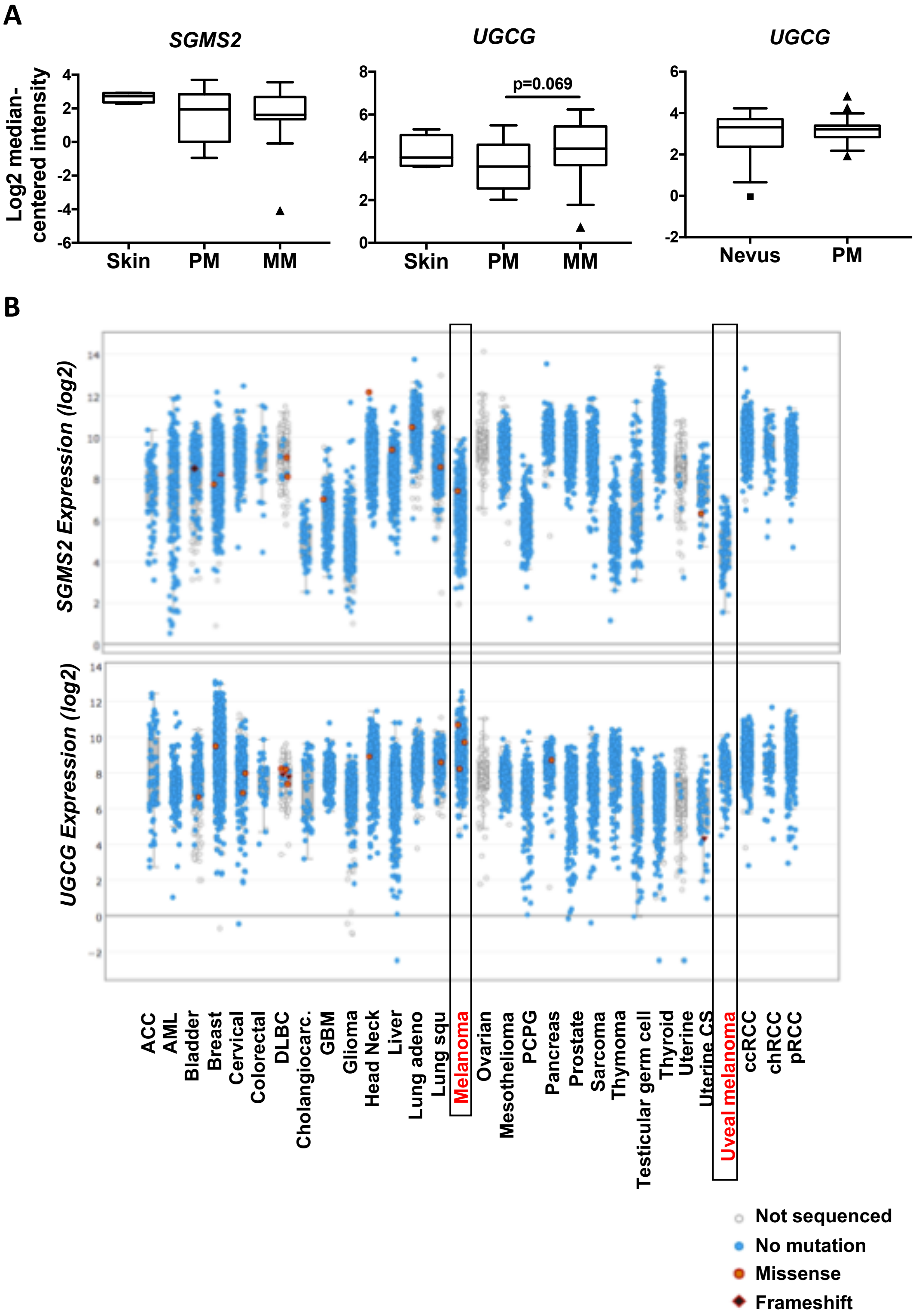

Supplementary Figure 1: Expression of SMS1, SMS2 and GCS in cancers. A, SMS2 (encoded by *SGMS2*) and GCS (encoded by *UGCG*) expression was analysed in the Ricker (left and middle panels) and Talantov (right panel) cohorts. Of note, no *SGMS2* probe was found in Talantov's cohort and both *SGMS2* and *UGCG* probes were missing in Haqq's cohort. **B,** The expression of *SGMS2* and *UGCG* was analysed in various cancer type cohorts from cbioportal. Missense and frameshift mutations are depicted.

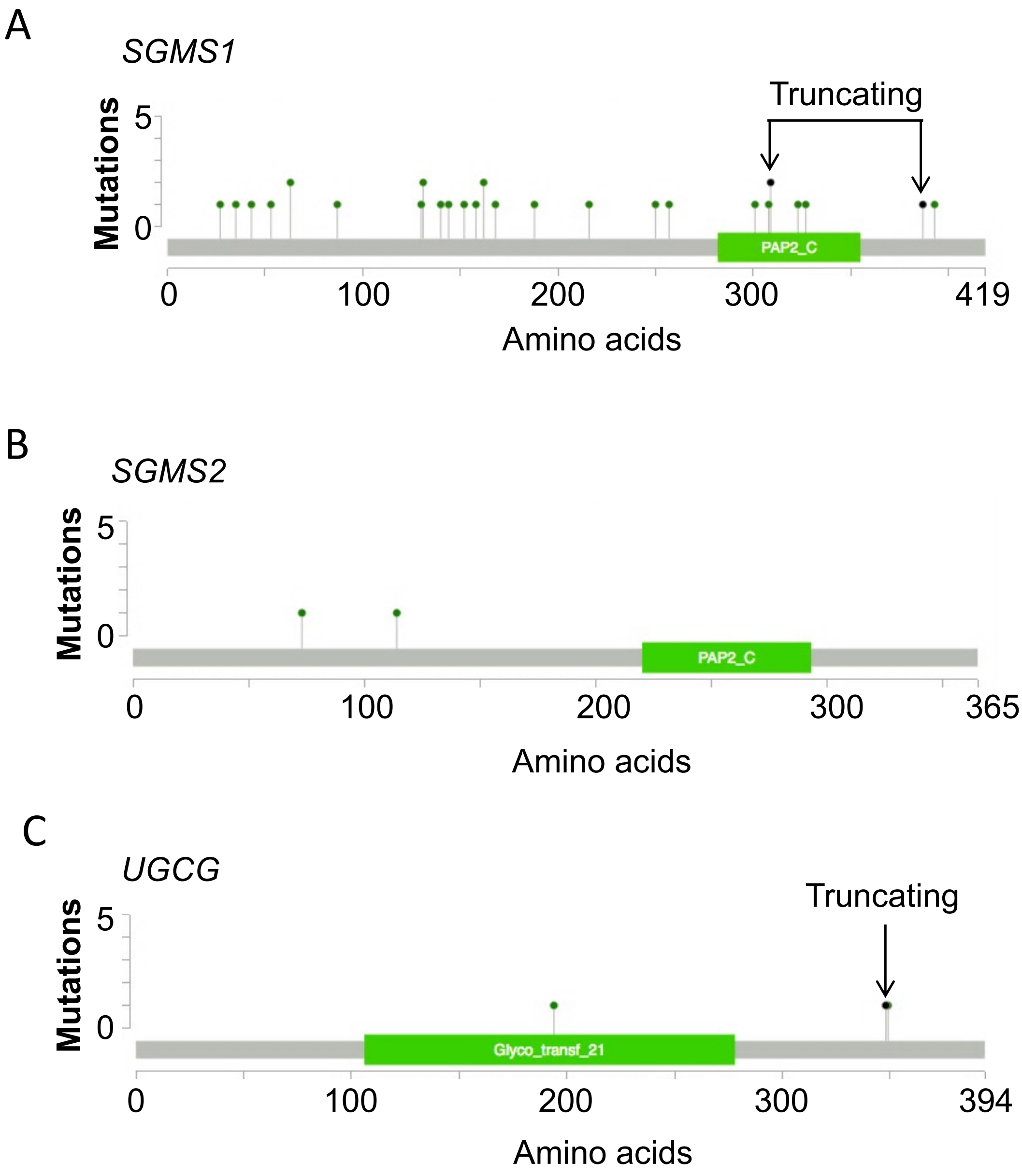

Supplementary Figure 2: Mutation mapping on *SGMS1*, *SGMS2* and *UGCG* in melanoma samples. Missense and truncated (as indicated by arrows) mutations in melanoma samples on *SGMS1* (A), *SGMS2* (B) and *UGCG* (C) were mapped using cbioportal.

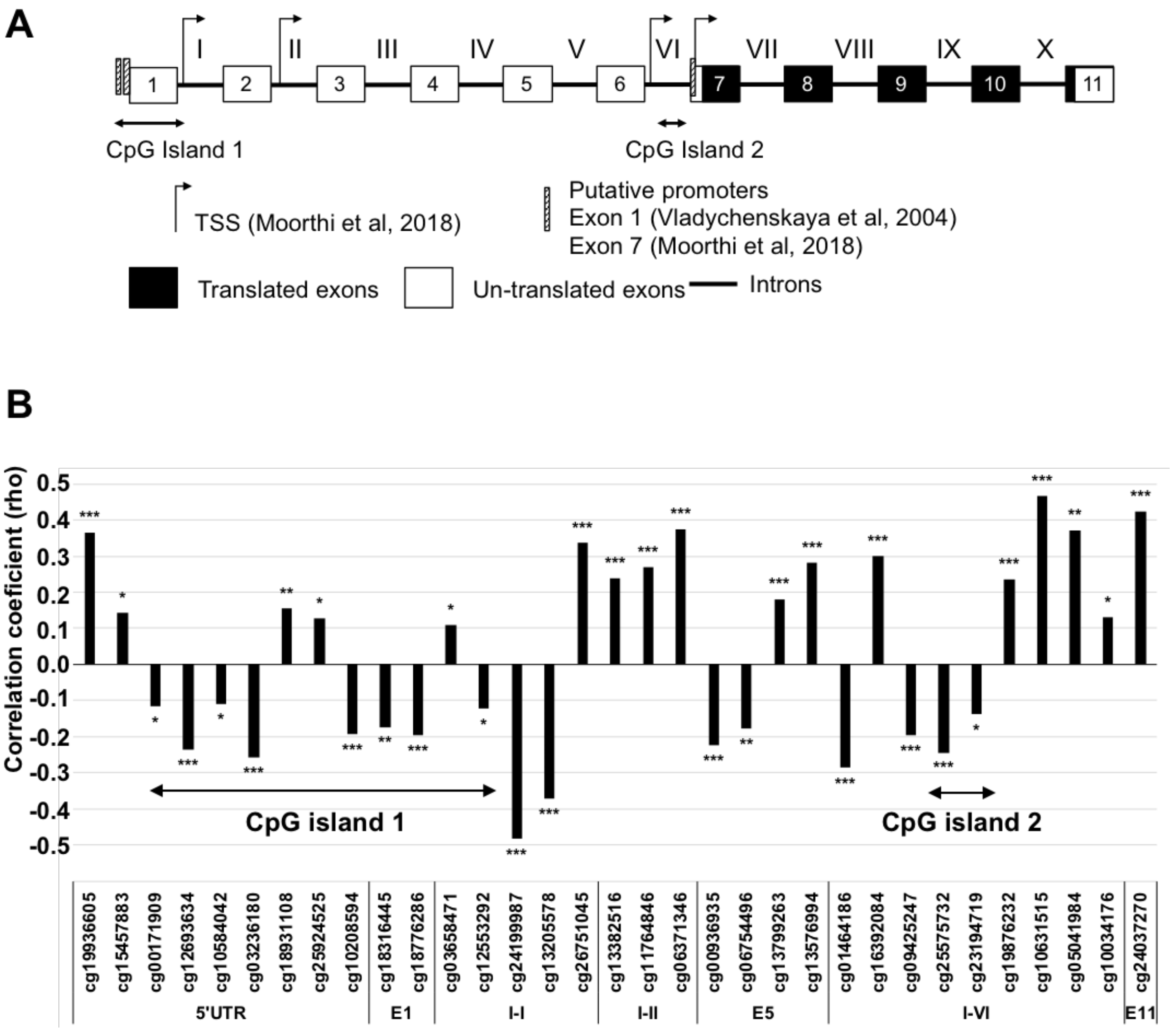

Supplementary Figure 3: Impact of CpG methylation on the expression of the *SMPD3* gene in metastatic melanoma. **A,** Two CpG islands of 256 (island 1) and 52 (island 2) CpG are located over the 5'UTR as indicated on the *SGMS1* gene. **B,** Correlation between *SGMS1* expression and methylation status of *SGMS1* CpGs in samples from patient with metastatic disease.

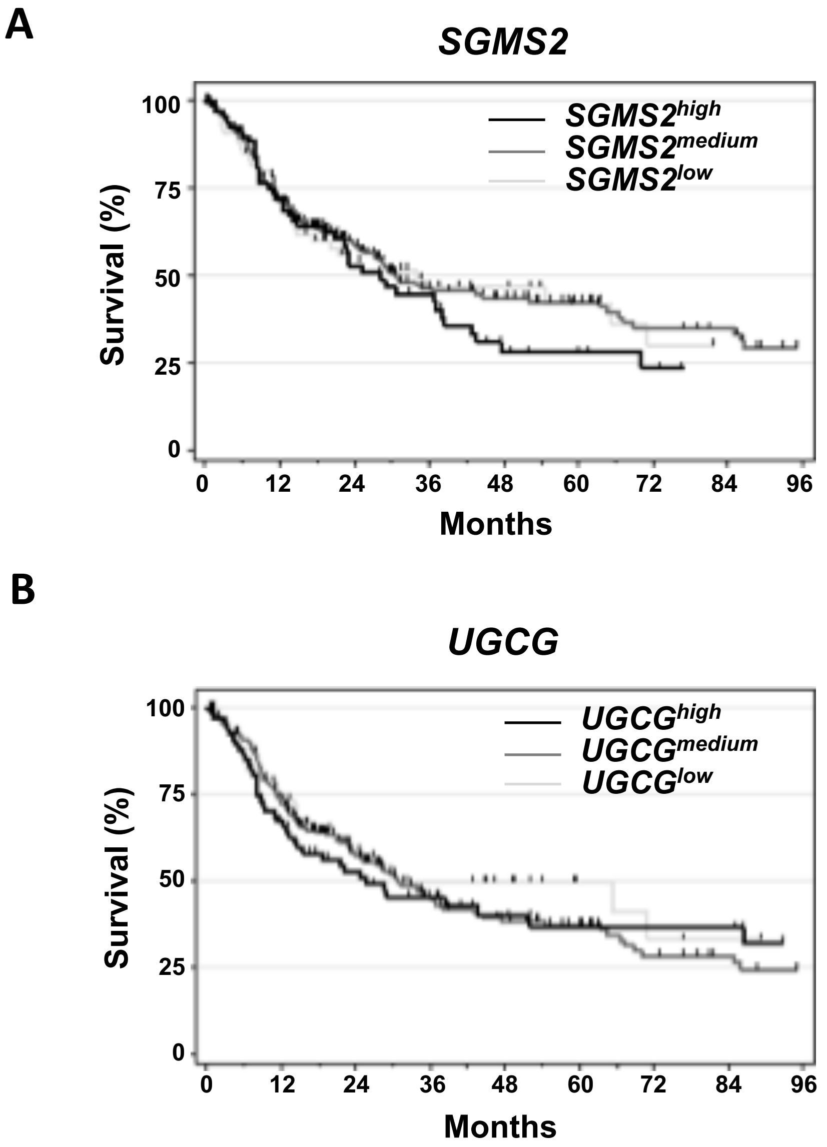

Supplementary Figure 4: *SGMS2* and *UGCG* expression levels in melanoma samples do not change overall survival of metastatic melanoma patients. Analysis of overall survival in metastatic melanoma patients from the TCGA melanoma cohort (n=342), exhibiting low (20th percentile; n=68), medium (between the 20th and 80th percentile) and high (80th percentile; n=68) *SGMS2* (A) and *UGCG* (B) expression in melanoma samples. Cox model: *SGMS2*^{low} (Reference), *SGMS2*^{medium}: HR= 0.97 [95% C.I.=.66;1.42] p=0.860; *SMS2*^{high}: HR= 1.19 [95% C.I..76;1.86] p=0.457 (A). Cox model: *UGCG*^{low} (Reference), *UGCG*^{medium}: HR= 1.15 [95% C.I.=.77;1.71] p=0.494; *UGCG*^{high}: HR= 1.16 [95% C.I..72;1.86] p=0.538 (B).
